# Supplementary material for: Seasonal Variation in Gut Microbiota Related to Diet in Fejervarya limnocharis
Source: Animals (Basel). 2021 May 13;11(5):1393. doi: 10.3390/ani11051393 (PMC8153623; doi:10.3390/ani11051393)
Supplement: Supplementary file 1 [file animals-11-01393-s001.zip › animals-1154089-supplementary.pdf]

Table S1. Sampling information of 170 individuals in *Fejervarya limnocharis* among different seasons.

Seasons	N	Temperature/℃	Water temperature/℃	Humidity	pH	Date
Spring	40	19.0	19.8	69.00%	7.21	04.08~04.12
Summer	40	27.0	29.0	78.80%	7.26	07.01~07.05
Autumn	45	22.5	23.2	71.90%	7.70	09.23~09.28
Pre-hibernation	45	16.3	16.4	83.10%	7.5	11.04~11.15

Table S2. Seasonal difference of alpha diversity index in frog gut microbial communities. C: Spring; X: Summer; Q: Autumn; D: Pre-hibernation.

Alpha diversity	Tax	Difference	Pvalue	LCL	UCL
ACE	C - D	33.278	0.000	16.081	50.475
	C - Q	39.530	0.000	21.707	57.354
	C - X	-22.454	0.013	-40.050	-4.857
	D - Q	6.252	0.477	-11.064	23.569
	D - X	-55.732	0.000	-72.815	-38.649
	Q - X	-61.984	0.000	-79.698	-44.271
Chao 1	C - D	30.203	0.001	12.759	47.647
	C - Q	37.648	0.000	19.568	55.727
	C - X	-23.165	0.011	-41.014	-5.316
	D - Q	7.445	0.404	-10.120	25.010
	D - X	-53.368	0.000	-70.696	-36.040
	Q - X	-60.813	0.000	-78.781	-42.846
Good coverage	C - D	-41.991	0.000	-58.564	-25.417
	C - Q	-42.902	0.000	-60.079	-25.725
	C - X	19.657	0.023	2.699	36.615
	D - Q	-0.911	0.914	-17.600	15.777
	D - X	61.648	0.000	45.185	78.111
	Q - X	62.559	0.000	45.488	79.630
Observed taxa	C - D	21.772	0.017	3.894	39.650
	C - Q	33.034	0.001	14.505	51.564
	C - X	-26.383	0.005	-44.676	-8.090
	D - Q	11.263	0.218	-6.739	29.265
	D - X	-48.155	0.000	-65.913	-30.396
	Q - X	-59.417	0.000	-77.832	-41.003
Shannon	C - D	-32.290	0.001	-50.766	-13.814
	C - Q	-11.746	0.228	-30.895	7.403
	C - X	-52.554	0.000	-71.459	-33.648
	D - Q	20.544	0.031	1.940	39.149
	D - X	-20.264	0.031	-38.617	-1.910
	Q - X	-40.808	0.000	-59.839	-21.777

Simpson	C - D	-52.626	0.000	-70.099	-35.153
	C - Q	-36.094	0.000	-54.204	-17.985
	C - X	-64.435	0.000	-82.314	-46.556
	D - Q	16.532	0.065	-1.063	34.126
	D - X	-11.809	0.181	-29.166	5.548
	Q - X	-28.341	0.002	-46.339	10.343

Table S3. The alpha diversity of gut microbiota in all *Fejervarya limnocharis* samples.

Sample name	Observed species	Shannon	Simpson	Chao1	ACE	Goods coverage	PD whole tree
C.M.01	1183	3.819	0.763	1887.979	2126.266	0.984	123.064
C.M.02	872	3.407	0.646	1229.408	1323.678	0.990	100.646
C.M.03	1104	5.654	0.918	1537.680	1592.674	0.988	117.320
C.M.04	2070	8.200	0.983	2451.369	2543.252	0.985	188.127
C.M.05	1113	4.219	0.865	1801.204	1936.978	0.984	122.858
C.M.06	394	3.297	0.801	595.397	616.535	0.995	57.461
C.M.07	1102	4.923	0.800	1321.430	1451.641	0.990	127.701
C.M.08	1769	6.009	0.862	2685.347	2731.196	0.980	163.304
C.M.09	1726	6.182	0.939	2514.331	2666.821	0.980	161.885
C.M.10	1328	6.489	0.964	1864.250	1947.091	0.986	126.139
C.M.11	1612	6.311	0.937	2045.128	2132.880	0.985	163.844
C.M.12	1014	3.654	0.660	1391.680	1501.865	0.988	104.143
C.M.13	1664	6.517	0.942	1964.333	2084.638	0.987	154.336
C.M.14	632	4.710	0.904	989.500	1038.523	0.992	76.122
C.M.15	1400	5.624	0.858	1698.009	1863.910	0.987	136.079
C.M.16	2511	7.120	0.944	3779.884	3851.150	0.970	226.953
C.M.17	812	3.152	0.600	1254.346	1324.723	0.990	97.796
C.M.18	1402	5.493	0.838	1636.064	1705.192	0.989	136.080
C.M.19	1086	6.733	0.975	1381.224	1459.007	0.990	108.701
C.M.20	862	3.846	0.704	1084.357	1200.873	0.991	97.463
C.M.21	1103	3.759	0.679	1564.551	1726.076	0.987	118.510
C.M.22	1747	6.431	0.925	2789.454	2955.102	0.978	177.541
C.F.01	1683	6.705	0.944	2498.282	2600.318	0.981	153.148
C.F.02	723	3.846	0.729	1077.449	1191.809	0.991	77.651
C.F.03	1437	4.610	0.729	1733.684	1854.230	0.987	147.406
C.F.04	2373	8.106	0.967	3213.453	3247.441	0.977	204.022
C.F.05	2669	7.163	0.930	4063.647	4255.149	0.967	236.046
C.F.06	1703	5.377	0.873	2504.848	2694.290	0.979	177.900
C.F.07	991	4.375	0.811	1374.580	1490.593	0.989	108.278
C.F.08	901	4.208	0.803	1158.493	1238.433	0.991	104.141
C.F.09	1413	4.943	0.784	1960.011	2052.649	0.985	146.582
C.F.10	1430	5.136	0.770	1655.272	1759.829	0.989	138.837
C.F.11	1960	6.308	0.854	2818.991	2936.607	0.979	169.350
C.F.12	1419	5.595	0.842	1886.610	1933.647	0.986	142.929
C.F.13	1667	4.687	0.707	2327.778	2370.171	0.982	164.635
C.F.14	1365	5.935	0.938	1978.804	2092.626	0.984	143.551
C.F.15	648	4.736	0.863	913.290	1013.138	0.993	74.877
C.F.16	1941	6.083	0.850	2286.548	2402.238	0.985	176.805
C.F.17	1381	4.979	0.796	1874.011	1959.900	0.985	148.810
X.M.01	1399	6.278	0.938	1864.878	1965.324	0.986	138.025

X.M.02	869	6.019	0.941	1207.450	1233.979	0.992	83.201
X.M.03	1672	6.161	0.949	2227.309	2364.434	0.983	166.077
X.M.04	1856	8.189	0.989	2463.020	2502.799	0.983	168.599
X.M.05	1603	6.446	0.943	2072.678	2177.408	0.985	163.553
X.M.06	1141	4.818	0.875	1709.519	1862.845	0.986	123.433
X.M.07	2620	8.38	0.983	3128.476	3197.381	0.980	226.013
X.M.08	1179	7.006	0.978	1551.216	1623.249	0.989	118.872
X.M.09	2626	8.835	0.993	3699.817	3765.664	0.973	236.411
X.M.10	1369	6.377	0.957	2115.405	2209.402	0.983	138.005
X.M.11	1302	6.381	0.964	1811.012	1957.503	0.986	141.149
X.M.12	1151	7.125	0.978	1735.267	1866.807	0.987	116.550
X.M.13	1546	7.001	0.970	1923.582	2012.286	0.986	160.358
X.M.14	2074	8.107	0.984	2460.150	2553.019	0.984	186.909
X.M.15	2402	7.358	0.962	3184.892	3308.848	0.976	213.690
X.M.16	1503	6.989	0.974	1805.349	1881.907	0.988	178.648
X.M.17	1870	6.251	0.877	2254.877	2358.662	0.984	186.751
X.M.18	3523	9.704	0.996	4517.469	4614.933	0.968	297.004
X.M.19	3828	9.983	0.997	5047.491	5162.126	0.964	301.611
X.M.20	531	3.278	0.779	697.260	768.786	0.994	91.732
X.M.21	1198	4.577	0.855	1753.736	1895.967	0.985	135.876
X.M.22	4466	10.659	0.999	5724.560	5813.995	0.961	355.479
X.M.23	1894	7.319	0.977	2700.382	2837.557	0.979	179.407
X.M.24	2835	9.201	0.994	3107.190	3244.099	0.983	248.382
X.M.25	1477	3.713	0.616	2015.367	2110.355	0.984	142.190
X.M.26	4514	10.652	0.998	5703.150	5746.013	0.961	360.754
X.M.27	1954	8.123	0.986	2276.450	2465.697	0.985	193.450
X.F.01	2088	8.100	0.987	2697.173	2745.137	0.981	192.298
X.F.02	2106	7.734	0.982	3083.372	3154.161	0.977	201.906
X.F.03	1826	8.478	0.992	2294.366	2333.776	0.985	168.343
X.F.04	1450	6.162	0.921	1981.546	2016.696	0.985	140.529
X.F.05	1772	6.263	0.944	2269.774	2420.768	0.982	160.940
X.F.06	1136	6.717	0.973	1630.299	1675.426	0.988	112.466
X.F.07	838	5.615	0.941	1114.129	1247.148	0.991	92.901
X.F.08	2112	8.374	0.99	2535.141	2599.432	0.984	223.710
X.F.09	2534	8.419	0.967	3109.846	3175.128	0.980	228.619
X.F.10	1163	4.200	0.823	1505.265	1632.761	0.988	152.273
X.F.11	1956	6.862	0.959	3054.919	3162.793	0.976	175.118
X.F.12	2074	7.581	0.974	2811.490	2952.157	0.979	191.307
X.F.13	1408	5.185	0.883	2090.442	2274.917	0.982	143.105
Q.M.01	977	5.776	0.945	1272.029	1302.410	0.991	94.971
Q.M.02	2023	8.184	0.982	2328.400	2452.717	0.986	173.549
Q.M.03	705	5.500	0.948	1003.819	1095.670	0.992	79.254
Q.M.04	750	5.752	0.950	908.988	991.948	0.993	76.960
Q.M.05	1096	4.825	0.901	1695.282	1772.519	0.986	122.043

Q.M.06	2416	7.329	0.969	3157.965	3288.867	0.975	219.646
Q.M.07	972	3.972	0.816	1353.031	1445.375	0.989	105.727
Q.M.08	2226	6.810	0.946	2663.506	2666.951	0.983	214.409
Q.M.09	502	5.727	0.960	588.835	619.934	0.997	50.326
Q.M.10	706	5.976	0.938	946.191	958.937	0.994	67.236
Q.M.11	1106	6.504	0.971	1521.400	1603.819	0.989	103.906
Q.M.12	724	4.386	0.745	868.467	906.149	0.994	77.837
Q.M.13	625	6.025	0.963	805.430	850.203	0.994	72.017
Q.M.14	1050	5.656	0.920	1399.116	1431.488	0.99	99.634
Q.M.15	700	6.674	0.976	937.406	969.922	0.994	67.272
Q.M.16	1633	7.106	0.966	1843.376	1866.229	0.99	162.812
Q.M.17	874	5.020	0.910	1042.600	1146.719	0.992	92.611
Q.M.18	2305	7.070	0.940	2989.556	3053.467	0.978	205.583
Q.M.19	1024	6.393	0.969	1470.874	1406.369	0.99	96.553
Q.M.20	637	5.678	0.955	860.009	906.767	0.994	66.562
Q.M.21	787	4.540	0.841	941.036	1000.218	0.994	82.669
Q.F.01	766	5.800	0.952	1062.833	1109.520	0.992	84.209
Q.F.02	540	3.648	0.670	688.193	705.938	0.995	59.401
Q.F.03	1136	3.573	0.723	1755.118	1901.285	0.985	129.100
Q.F.04	734	6.284	0.970	949.821	1021.814	0.993	71.456
Q.F.05	684	6.308	0.967	791.341	823.011	0.995	65.710
Q.F.06	553	4.801	0.905	787.611	822.813	0.994	63.211
Q.F.07	663	5.715	0.955	924.604	968.865	0.993	77.058
Q.F.08	2154	8.290	0.986	2398.837	2407.261	0.988	221.937
Q.F.09	842	6.098	0.965	1441.234	1559.639	0.989	89.089
Q.F.10	471	3.958	0.801	710.718	757.015	0.995	54.691
Q.F.11	549	4.855	0.925	772.441	820.503	0.994	72.150
Q.F.12	429	4.878	0.930	447.025	475.100	0.998	48.048
Q.F.13	2075	7.277	0.973	2575.458	2649.313	0.982	197.813
Q.F.14	431	5.228	0.943	556.304	581.005	0.996	53.031
Q.F.15	2094	6.065	0.919	2444.041	2551.755	0.984	206.306
Q.F.16	501	4.267	0.846	624.759	679.018	0.995	61.984
Q.F.17	1411	6.103	0.946	2117.255	2278.475	0.983	134.286
D.M.01	1407	5.580	0.832	1808.278	1889.808	0.987	135.834
D.M.02	802	6.615	0.978	1030.444	1091.732	0.993	86.403
D.M.03	801	5.915	0.957	1045.079	1099.688	0.992	91.698
D.M.04	1296	6.356	0.952	1611.918	1695.646	0.988	132.598
D.M.05	1233	7.141	0.978	1603.435	1663.376	0.989	118.375
D.M.06	938	5.489	0.927	1197.508	1233.715	0.991	104.667
D.M.07	706	4.876	0.873	955.160	1024.165	0.993	75.815
D.M.08	778	6.144	0.965	953.000	1010.552	0.994	82.325
D.M.09	816	6.399	0.965	1051.812	1133.797	0.992	81.573
D.M.10	722	5.743	0.951	994.826	1019.707	0.993	78.630
D.M.11	810	6.338	0.961	1025.555	1034.519	0.994	77.073

D.M.12	784	6.359	0.962	954.507	1012.489	0.994	77.350
D.M.13	1079	6.194	0.949	1407.197	1482.863	0.990	102.415
D.M.14	784	6.919	0.977	1003.204	1023.037	0.994	69.538
D.M.15	843	5.195	0.910	1190.211	1232.924	0.991	86.238
D.M.16	1200	7.480	0.979	1335.937	1349.226	0.994	120.237
D.M.17	1426	8.575	0.992	1612.107	1585.235	0.993	151.614
D.M.18	1415	6.820	0.963	1788.338	1919.136	0.987	131.153
D.M.19	926	5.465	0.920	1167.684	1214.788	0.992	95.090
D.M.20	1501	6.974	0.969	1928.812	1931.283	0.987	145.699
D.M.21	935	5.642	0.941	1266.373	1343.521	0.990	97.949
D.M.22	1867	7.538	0.970	2321.819	2293.693	0.986	179.551
D.M.23	2096	7.994	0.982	2822.814	2789.563	0.981	184.540
D.M.24	1051	4.597	0.829	1355.481	1482.773	0.989	113.833
D.M.25	733	6.185	0.967	941.496	1007.946	0.993	74.547
D.M.27	1436	6.121	0.907	1838.230	1910.127	0.986	140.583
D.M.28	751	5.249	0.921	1000.407	1056.804	0.993	84.150
D.M.29	895	6.279	0.945	1082.280	1122.016	0.993	83.384
D.M.30	990	6.285	0.958	1287.179	1259.968	0.991	105.607
D.M.31	857	4.767	0.862	1227.875	1309.425	0.990	88.783
D.M.32	1726	7.189	0.966	2111.031	2158.685	0.986	152.591
D.M.33	1792	7.161	0.973	2213.557	2290.229	0.984	163.480
D.M.34	1537	8.692	0.992	1832.042	1754.468	0.992	160.251
D.M.35	1092	7.003	0.975	1309.000	1327.203	0.992	107.514
D.M.36	900	5.966	0.944	1141.882	1136.499	0.993	95.498
D.M.37	745	5.514	0.943	1012.022	1080.953	0.992	83.557
D.M.38	788	6.003	0.945	942.878	979.221	0.994	77.628
D.M.39	1826	7.719	0.983	2278.003	2365.384	0.984	167.546
D.M.40	1971	7.666	0.985	2524.459	2574.098	0.982	189.511
D.M.41	2136	8.823	0.991	2397.113	2370.929	0.989	198.677
D.M.42	517	4.476	0.862	660.000	665.696	0.996	55.323
D.F.01	692	6.024	0.957	867.290	942.500	0.994	71.715
D.F.02	671	5.932	0.951	808.462	873.630	0.995	70.171
D.F.03	891	4.748	0.810	1210.168	1313.235	0.991	94.580

Table S4. Inter-and intra-species differences in the microbiota among seasons. C: Spring; X: Summer; Q: Autumn; D: Pre-hibernation.

**Group**	**R-value**	***P*-value**
Q - X	0.2698	0.001
C - X	0.6255	0.001
C - Q	0.6365	0.001
D - X	0.3811	0.001
D - Q	0.1984	0.001
D - C	0.8008	0.001

Table S5. Summary of *t*-test in different levels with the significant thresholds ($P < 0.05$). C: Spring; X: Summer; Q: Autumn; D: Pre-hibernation.

Tax	Phylum	Class	Order	Family	Genus	Species
C-X	14	23	41	76	213	177
C-Q	9	17	38	75	199	153
C-D	16	21	43	82	211	160
X-Q	8	15	43	94	209	153
X-D	9	14	40	79	207	157
Q-D	7	7	16	33	77	44

Table S6. Species differences between groups below the level of phylum. C: Spring; X: Summer; Q: Autumn; D: Pre-hibernation.

C-D	C-Q	C-X	Q-D	X-D	X-Q
Firmicutes	Firmicutes	Proteobacteria	Bacteroidetes	Firmicutes	Firmicutes
Proteobacteria	Proteobacteria	Bacteroidetes	Spirochaetes	Proteobacteria	Actinobacteria
Bacteroidetes	Cyanobacteria	Tenericutes	Deferribacteres	Actinobacteria	Planctomycetes
Cyanobacteria	Actinobacteria	Euryarchaeota	Deinococcus thermus	Spirochaetes	Chloroflexi
Actinobacteria	Tenericutes	Planctomycetes	Gracilibacteria	Planctomycetes	Fibrobacteres
Tenericutes	Spirochaetes	Gemmatimonadetes	Candidatus Moranbacteria	Deferribacteres	Thermotogae
Spirochaetes	Euryarchaeota	Chloroflexi	Atribacteria	Gemmatimonadetes	Candidatus peregrinibacteria
Euryarchaeota	Deferribacteres	Rokubacteria		Chloroflexi	Atribacteria
Deferribacteres	Atribacteria	Nitrospirae		Fibrobacteres	
Rokubacteria		Deinococcus thermos			
Nitrospirae		Fibrobacteres			
Deinococcus thermos		Elusimicrobia			
Gracilibacteria		Kiritimatiellaeota			
Kiritimatiellaeota		Candidatus peregrinibacteria			
Candidatus Moranbacteria					
Dadabacteria					

Table S7. Significantly different species between seasons below the level of genus. The top 10 of extremely significant difference was shown in this table (*P* < 0.01); C: Spring; X: Summer; Q: Autumn; D: Pre-hibernation.

Tax	Number	Top 10
C-X	213	*Stenotrophomonas; Delftia; Megasphaera; Fastidiosipila; Neisseria; Commensalibacter; Gardnerella; Unidentified_Actinobacteria; Halomona*s; *Coxiella;*
C-Q	199	*Stenotrophomonas; Hydrogenispora; Fastidiosipila; Hyphomicrobium; Blastococcus; Desulfovibrio; Anaerobacter; Paenarthrobacter; Solibacillus; Antricoccus;*
C-D	211	*Stenotrophomonas; Unidentified_Clostridiales; Alistipes; Succinivibrio; Nocardioides; Oscillibacter; Brevinema; Bacteroides; Microlunatus; Blastococcus;*
X-Q	209	*Anaerobacterium; Desulfosporosinus; Conexibacter;* Hydrogenispora; *Nordella; Sporomusa; Pajaroellobacter; Sporacetigenium; Streptomyces; Rhizocola;*
X-D	207	*Succinivibrio; Desulfosporosinus; Brevinema; Unidentified_Actinobacteria; Nocardioides; Conexibacter; Marmoricola; Sporomusa; Mucispirillum; Candidatus_Alysiosphaera;*
Q-D	77	*Succinivibrio; Alistipes; Coxiella; Brevinema; Alcaligenes; Mucispirillum; Actinomycetospora; Virgisporangium; Gemmobacter; Robinsoniella;*

Table S8: Functional differences between different seasons. Level 1 and level 2 represent levels 1 and 2 of KEGG, respectively; C: Spring; X: Summer; Q: Autumn; D: Pre-hibernation; "+" means difference is significant (*P* < 0.05).

**Level 1**	**Level 2**	**C-D**	**C-Q**	**C-X**	**Q-D**	**X-D**	**X-Q**
Cellular processes	Cell growth and death	+	+	+		+	+
	Cellular community - prokaryotes	+	+	+		+	
	Cell motility		+			+	+
	Transport and catabolism	+	+	+	+	+	
Environmental information processing	Membrane transport	+	+	+			
	Signal transduction	+		+	+		+
	Signaling molecules and interaction	+			+		
Genetic information processing	Folding, sorting and degradation	+	+			+	+
	Replication and repair	+	+			+	+
	Transcription	+	+			+	+
	Translation		+				
Human diseases	Cancers	+	+	+	+		+
	Cardiovascular diseases	+	+	+			
	Endocrine and metabolic diseases	+	+	+	+	+	
	Immune diseases		+				
	Infectious diseases	+					
	Neurodegenerative diseases	+	+	+		+	
	Substance dependence	+	+			+	+
Metabolism	Amino acid metabolism	+	+			+	+
	Biosynthesis of other secondary metabolites	+	+	+	+		
	Carbohydrate metabolism	+	+	+	+		
	Energy metabolism		+	+	+	+	

	Enzyme families	+			+		+
	Glycan biosynthesis and metabolism	+			+	+	
	Lipid metabolism	+	+			+	+
	Metabolism of cofactors and vitamins					+	
	Metabolism of other amino acids	+	+			+	+
	Metabolism of terpenoids and polyketides	+	+			+	+
	Nucleotide metabolism	+	+	+		+	+
	Xenobiotics biodegradation and metabolism	+	+	+		+	+
Organismal Systems	Aging	+	+			+	+
	Circulatory system	+	+	+			
	Digestive system	+		+	+		+
	Endocrine system	+	+		+	+	+
	Environmental adaptation		+	+		+	+
	Excretory system	+	+			+	+
	Immune system	+	+				+
	Nervous system	+	+	+			
Unclassified	Cellular processes and signaling	+				+	
	Genetic information processing	+		+	+	+	+
	Metabolism						+
	Poorly characterized	+	+			+	+
	Viral protein family	+	+			+	+

Table S9. Stomach contents of *Fejervarya limnocharis* in farmlands. In the table, N1: number of prey, K: percentage of number of prey, N2: frequency of each prey item, P: percentage of frequency of each prey item.

Prey category	N_1	K	N_2	P
**Insecta**				
Orthoptera	20	5.36%	19	8.33%
Coleoptera	36	9.65%	34	14.91%
Hemiptera	18	4.83%	13	5.70%
Lepidoptera (Larvae)	16	4.29%	14	6.14%
Hymenoptera	24	6.43%	16	7.02%
Diptera	46	12.33%	21	9.21%
Blattaria	1	0.27%	1	0.44%
Dermaptera	12	3.22%	10	4.39%
Neuroptera	18	4.83%	1	0.44%
**Arachnida**				
Araneae	70	18.77%	30	13.16%
Acariforms	2	0.54%	1	0.44%
Ixodida	1	0.27%	1	0.44%
**Oligochaeta**				
Haplotaxida	8	2.14%	8	3.51%
**Malacostraca**				
Isopoda	52	13.94%	27	11.84%
**Chilopoda**				
Scolopendromorpha	32	8.58%	23	10.09%
**Gastropoda**				
Mesogastropoda	6	1.61%	3	1.32%
Stylommatophora	1	0.27%	1	0.44%
Tectibranchia	1	0.27%	1	0.44%
**Unidentified items**	9	2.41%	4	1.75%
**Total**	373	100.00%	228	100.00%

Table S10. The feeding habits of *Fejervarya limnocharis* in different seasons (N=114). In the table, N_1 represents the amount of food eaten by the *F. limnocharis* in each classification; K is for food percentage; N_2 represents the occurrence frequency of each classification in the stomachs of *F. limnocharis*; P is for the percentage of frequency.

Food contents	Spring				Summer				Autumn				Pre-hibernation			
	N_1	K	N_2	P	N_1	K	N_2	P	N_1	K	N_2	P	N_1	K	N_2	P
**Insecta**																
Orthoptera	3	2.14%	3	3.19%	2	1.74%	2	4.08%	8	10.26%	8	15.38%	7	17.50%	6	18.18%
Coleoptera	19	13.57%	17	18.09%	5	4.35%	5	10.20%	7	8.97%	7	13.46%	5	12.50%	5	15.15%
Hemiptera	8	5.71%	5	5.32%	4	3.48%	3	6.12%	6	7.69%	5	9.62%				
Lepidoptera	11	7.86%	9	9.57%					3	3.85%	3	5.77%	2	5.00%	2	6.06%
Hymenoptera	8	5.71%	6	6.38%	9	7.83%	4	8.16%	5	6.41%	4	7.69%	2	5.00%	2	6.06%
Diptera	36	25.71%	15	15.96%	3	2.61%	3	6.12%					7	17.50%	3	9.09%
**Blattaria**													1	2.50%	1	3.03%
Dermaptera	10	7.14%	8	8.51%	2	1.74%	2	4.08%								
Neuroptera					18	15.65%	1	2.04%								
**Arachnida**																
Araneae	14	10.00%	11	11.70%	34	29.57%	9	18.37%	18	23.08%	7	13.46%	4	10.00%	3	9.09%
Acariforms	2	1.43%	1	1.06%												
Ixodida									1	1.28%	1	1.92%				
**Oligochaeta**																
Haplotaxida					3	2.61%	3	6.12%	3	3.85%	3	5.77%	2	5.00%	2	6.06%
**Malacostraca**																
Isopoda	4	2.86%	2	2.13%	32	27.83%	14	28.57%	11	14.10%	6	11.54%	5	12.50%	5	15.15%
**Chilopoda**																

Scolopendromorpha	25	17.86%	17	18.09%	1	0.87%	1	2.04%	2	2.56%	2	3.85%	4	10.00%	3	9.09%
**Gastropoda**																
Mesogastropoda									5	6.41%	2	3.85%	1	2.50%	1	3.03%
Stylommatophora									1	1.28%	1	1.92%				
Tectibranchia					1	0.87%	1	2.04%								
**Unidentified items**					1	0.87%	1	2.04%	8	10.26%	3	5.77%				

Table S11. Comparison of food diversity and niche width in *Fejervarya limnocharis* among different seasons.

Item	Spring	Summer	Autumn	Pre-hibernation
Food diversity (Shannon-Weiner index)	2.118	1.765	2.304	1.765
Niche width (Simpson diversity index)	0.871	0.851	0.898	0.883

Table S12. Food diversity and niche widths between males and females in *Fejervarya limnocharis* among different seasons. H: Food diversity (Shannon-Weiner index); B: Niche width (Simpson diversity index). Due to the small number of females in pre-hibernation, for the sake of data reliability, the pre-hibernation feeding habits of males and females are not analyzed here.

**Seasons**	**H**		**B**	
	**Male**	**Female**	**Male**	**Female**
Spring	2.038	2.087	0.866	0.864
Summer	1.737	1.264	0.837	0.805
Autumn	2.112	2.311	0.881	0.889

Table S13. The type and number of food in *F. limnocharis* (N=114). No statistics on leaves, meat and food scraps. There were 147 solid stomachs (47 females), and 114 identified species (38 females). In the table, M is male; F is female; Orth, Cole, Hemi, Lepi, Hyme, Aran, Ixod, Hapl, Isop, Scol, Meso, Styl, Dipt, Derm, Blat, Acar, Tect, Neur and Unknow represent Orthoptera, Coleoptera, Hemiptera, Lepisoprera, Hymenoptera, Araneida, Ixodida, Haplotaxida, Isoptera, Scolopendromorpha, Mesogastropoda, Stylommatophora, Diptera, Dermaptera, Blattaria, Acariforms, Tectibranchia, Neuroptera and An unknown species, respectively.

Sample	Gender	Orth	Cole	Hemi	Lepi	Hyme	Aran	Ixod	Hapl	Isop	Scol	Meso	Styl	Dipt	Derm	Blat	Acar	Tect	Neur	Unknow
Aut01	M	1	0	0	0	0	0	0	0	0	0	0	0	0	0	0	0	0	0	0
Aut03	F	1	1	0	0	0	0	0	0	0	0	1	0	0	0	0	0	0	0	0
Aut04	M	1	0	0	0	0	0	0	0	0	0	0	0	0	0	0	0	0	0	0
Aut05	M	0	0	0	0	0	12	0	0	0	0	4	0	0	0	0	0	0	0	1
Aut06	M	0	0	0	0	1	0	0	1	0	0	0	0	0	0	0	0	0	0	0
Aut09	M	0	0	1	0	0	0	0	0	0	0	0	0	0	0	0	0	0	0	0
Aut10	F	0	0	0	1	0	0	1	0	0	1	0	0	0	0	0	0	0	0	0
Aut12	M	0	1	0	0	0	0	0	0	0	0	0	0	0	0	0	0	0	0	0
Aut13	M	0	0	0	0	1	0	0	0	0	0	0	0	0	0	0	0	0	0	0
Aut14	M	0	0	0	0	0	1	0	0	0	0	0	0	0	0	0	0	0	0	0
Aut15	F	0	0	2	0	0	0	0	0	0	0	0	1	0	0	0	0	0	0	0
Aut16	F	1	1	0	1	0	0	0	0	0	0	0	0	0	0	0	0	0	0	0
Aut17	F	0	1	0	0	0	0	0	0	1	0	0	0	0	0	0	0	0	0	4
Aut20	F	0	0	1	0	0	0	0	0	0	0	0	0	0	0	0	0	0	0	0
Aut21	M	1	0	1	0	0	0	0	0	0	0	0	0	0	0	0	0	0	0	0
Aut23	M	0	0	0	0	0	0	0	0	0	0	0	0	0	0	0	0	0	0	3
Aut24	F	1	0	0	0	0	1	0	0	0	0	0	0	0	0	0	0	0	0	0
Aut26	F	0	0	0	0	1	0	0	0	0	0	0	0	0	0	0	0	0	0	0
Aut29	M	0	0	0	0	0	1	0	0	0	0	0	0	0	0	0	0	0	0	0

Aut30	F	0	0	0	0	2	0	0	0	0	0	0	0	0	0	0	0	0	0	0
Aut31	M	0	1	0	0	0	1	0	0	1	0	0	0	0	0	0	0	0	0	0
Aut32	F	1	0	1	0	0	0	0	0	0	0	0	0	0	0	0	0	0	0	0
Aut34	F	0	1	0	0	0	0	0	0	0	0	0	0	0	0	0	0	0	0	0
Aut35	M	0	1	0	0	0	0	0	0	0	0	0	0	0	0	0	0	0	0	0
Aut36	M	0	0	0	0	0	0	0	0	1	0	0	0	0	0	0	0	0	0	0
Aut37	M	0	0	0	0	0	1	0	0	1	0	0	0	0	0	0	0	0	0	0
Aut38	F	0	0	0	0	0	1	0	1	0	0	0	0	0	0	0	0	0	0	0
Aut40	M	1	0	0	0	0	0	0	1	6	1	0	0	0	0	0	0	0	0	0
Spr01	M	0	0	0	0	0	0	0	0	0	2	0	0	0	1	0	0	0	0	0
Spr02	F	0	0	0	0	0	1	0	0	0	0	0	0	0	0	0	0	0	0	0
Spr03	M	0	1	0	1	0	0	0	0	0	1	0	0	6	0	0	0	0	0	0
Spr05	F	0	2	0	2	0	1	0	0	0	1	0	0	0	0	0	0	0	0	0
Spr06	M	0	1	0	0	0	0	0	0	0	0	0	0	1	0	0	2	0	0	0
Spr07	F	0	0	0	0	0	1	0	0	0	1	0	0	0	0	0	0	0	0	0
Spr08	F	0	1	0	1	1	0	0	0	0	0	0	0	1	0	0	0	0	0	0
Spr09	F	0	1	1	2	1	3	0	0	3	0	0	0	1	0	0	0	0	0	0
Spr11	M	0	0	0	0	0	0	0	0	0	0	0	0	2	0	0	0	0	0	0
Spr12	F	0	0	0	0	0	0	0	0	0	1	0	0	5	0	0	0	0	0	0
Spr13	M	0	0	0	1	0	0	0	0	0	0	0	0	0	0	0	0	0	0	0
Spr14	M	0	0	1	0	0	0	0	0	0	0	0	0	0	0	0	0	0	0	0
Spr15	F	1	0	1	0	0	2	0	0	0	2	0	0	1	0	0	0	0	0	0
Spr16	M	0	1	0	0	0	0	0	0	0	0	0	0	0	0	0	0	0	0	0
Spr17	F	1	2	0	0	0	1	0	0	0	1	0	0	0	3	0	0	0	0	0
Spr18	M	0	0	0	1	0	1	0	0	0	3	0	0	1	0	0	0	0	0	0
Spr20	F	0	1	0	0	0	0	0	0	0	1	0	0	0	0	0	0	0	0	0

Spr21	F	0	1	0	0	0	0	0	0	0	1	0	0	0	1	0	0	0	0	0
Spr22	M	0	0	0	0	0	0	0	0	0	1	0	0	1	0	0	0	0	0	0
Spr23	M	0	0	0	0	0	1	0	0	0	4	0	0	0	0	0	0	0	0	0
Spr24	M	0	0	0	0	0	0	0	0	0	0	0	0	0	1	0	0	0	0	0
Spr25	F	1	1	0	0	0	0	0	0	0	0	0	0	0	0	0	0	0	0	0
Spr26	M	0	1	0	0	3	0	0	0	0	0	0	0	1	0	0	0	0	0	0
Spr27	F	0	0	0	0	1	0	0	0	0	0	0	0	1	1	0	0	0	0	0
Spr28	F	0	1	3	0	0	0	0	0	0	0	0	0	10	0	0	0	0	0	0
Spr29	M	0	1	1	0	0	1	0	0	0	0	0	0	0	0	0	0	0	0	0
Spr30	M	0	0	0	0	0	0	0	0	0	0	0	0	0	1	0	0	0	0	0
Spr31	M	0	0	0	1	1	0	0	0	1	1	0	0	0	0	0	0	0	0	0
Spr33	F	0	1	0	0	0	0	0	0	0	2	0	0	0	1	0	0	0	0	0
Spr34	F	0	1	0	0	0	0	0	0	0	0	0	0	3	0	0	0	0	0	0
Spr35	F	0	0	0	0	0	1	0	0	0	0	0	0	0	0	0	0	0	0	0
Spr36	M	0	0	0	1	0	1	0	0	0	1	0	0	1	0	0	0	0	0	0
Spr37	F	0	1	0	0	0	0	0	0	0	1	0	0	0	0	0	0	0	0	0
Spr38	M	0	0	0	1	1	0	0	0	0	1	0	0	0	0	0	0	0	0	0
Spr39	M	0	1	0	0	0	0	0	0	0	0	0	0	0	1	0	0	0	0	0
Spr40	M	0	0	0	0	0	0	0	0	0	0	0	0	1	0	0	0	0	0	0
Sum01	F	0	0	0	0	0	1	0	0	0	0	0	0	0	0	0	0	0	0	0
Sum02	F	0	0	0	0	0	0	0	0	0	0	0	0	0	1	0	0	0	0	0
Sum03	F	0	0	0	0	0	0	0	0	3	0	0	0	0	0	0	0	0	0	0
Sum04	M	0	1	2	0	0	1	0	0	2	0	0	0	1	1	0	0	0	0	0
Sum06	F	0	1	0	0	6	0	0	0	0	0	0	0	0	0	0	0	0	0	0
Sum08	M	0	0	0	0	0	0	0	0	2	0	0	0	0	0	0	0	0	0	0
Sum09	M	1	0	0	0	1	0	0	0	2	0	0	0	0	0	0	0	0	0	0

Sum10	F	0	0	0	0	0	25	0	0	1	0	0	0	0	0	0	0	0	0	0
Sum11	M	0	1	0	0	0	0	0	0	0	0	0	0	0	0	0	0	0	0	0
Sum12	F	0	0	0	0	0	1	0	1	0	0	0	0	0	0	0	0	0	0	0
Sum14	M	0	0	1	0	0	0	0	0	0	0	0	0	0	0	0	0	0	0	0
Sum15	M	0	0	0	0	1	2	0	0	0	0	0	0	0	0	0	0	0	0	0
Sum16	M	0	0	0	0	0	0	0	0	0	1	0	0	0	0	0	0	0	0	0
Sum17	F	0	0	0	0	0	1	0	0	0	0	0	0	0	0	0	0	0	0	0
Sum18	M	0	0	1	0	0	0	0	0	3	0	0	0	0	0	0	0	0	0	0
Sum21	F	0	1	0	0	0	0	0	0	1	0	0	0	0	0	0	0	0	18	0
Sum22	F	0	1	0	0	0	0	0	0	0	0	0	0	0	0	0	0	0	0	0
Sum23	M	0	0	0	0	0	0	0	0	5	0	0	0	0	0	0	0	0	0	0
Sum24	M	0	0	0	0	0	1	0	0	0	0	0	0	0	0	0	0	0	0	1
Sum25	F	1	0	0	0	0	0	0	1	0	0	0	0	0	0	0	0	0	0	0
Sum27	M	0	0	0	0	0	0	0	0	1	0	0	0	1	0	0	0	0	0	0
Sum29	M	0	0	0	0	0	0	0	0	5	0	0	0	0	0	0	0	0	0	0
Sum30	F	0	0	0	0	0	1	0	1	0	0	0	0	1	0	0	0	0	0	0
Sum32	M	0	0	0	0	0	0	0	0	1	0	0	0	0	0	0	0	0	0	0
Sum33	M	0	0	0	0	0	1	0	0	0	0	0	0	0	0	0	0	0	0	0
Sum34	M	0	0	0	0	0	0	0	0	3	0	0	0	0	0	0	0	0	0	0
Sum35	M	0	0	0	0	0	0	0	0	0	0	0	0	0	0	0	0	1	0	0
Sum36	M	0	0	0	0	0	0	0	0	2	0	0	0	0	0	0	0	0	0	0
Sum40	M	0	0	0	0	1	0	0	0	1	0	0	0	0	0	0	0	0	0	0
Win01	F	0	0	0	1	0	0	0	0	0	0	0	0	0	0	0	0	0	0	0
Win02	M	1	1	0	0	0	0	0	1	0	0	0	0	0	0	0	0	0	0	0
Win04	M	0	0	0	0	0	1	0	0	0	0	0	0	0	0	0	0	0	0	0
Win11	M	0	1	0	0	0	0	0	0	0	0	0	0	0	0	0	0	0	0	0

Win14	M	0	0	0	0	0	0	0	0	0	0	1	0	0	0	0	0	0	0	0
Win20	M	0	0	0	0	0	0	0	0	1	0	0	0	0	0	0	0	0	0	0
Win21	M	1	0	0	0	0	0	0	0	1	0	0	0	0	0	0	0	0	0	0
Win23	M	0	0	0	0	0	0	0	0	1	1	0	0	1	0	0	0	0	0	0
Win25	M	0	0	0	0	1	1	0	0	0	0	0	0	0	0	0	0	0	0	0
Win26	M	0	0	0	0	0	0	0	1	1	0	0	0	2	0	0	0	0	0	0
Win28	M	1	0	0	0	0	0	0	0	0	1	0	0	0	0	0	0	0	0	0
Win29	M	1	0	0	0	0	0	0	0	0	0	0	0	0	0	0	0	0	0	0
Win31	M	0	0	0	1	0	0	0	0	0	0	0	0	0	0	0	0	0	0	0
Win33	M	0	0	0	0	0	0	0	0	1	0	0	0	0	0	0	0	0	0	0
Win36	M	0	1	0	0	0	2	0	0	0	0	0	0	0	0	0	0	0	0	0
Win37	M	0	0	0	0	0	0	0	0	0	2	0	0	0	0	0	0	0	0	0
Win40	M	1	0	0	0	0	0	0	0	0	0	0	0	4	0	0	0	0	0	0
Win41	M	0	1	0	0	1	0	0	0	0	0	0	0	0	0	1	0	0	0	0
Win44	M	2	0	0	0	0	0	0	0	0	0	0	0	0	0	0	0	0	0	0
Win45	M	0	1	0	0	0	0	0	0	0	0	0	0	0	0	0	0	0	0	0

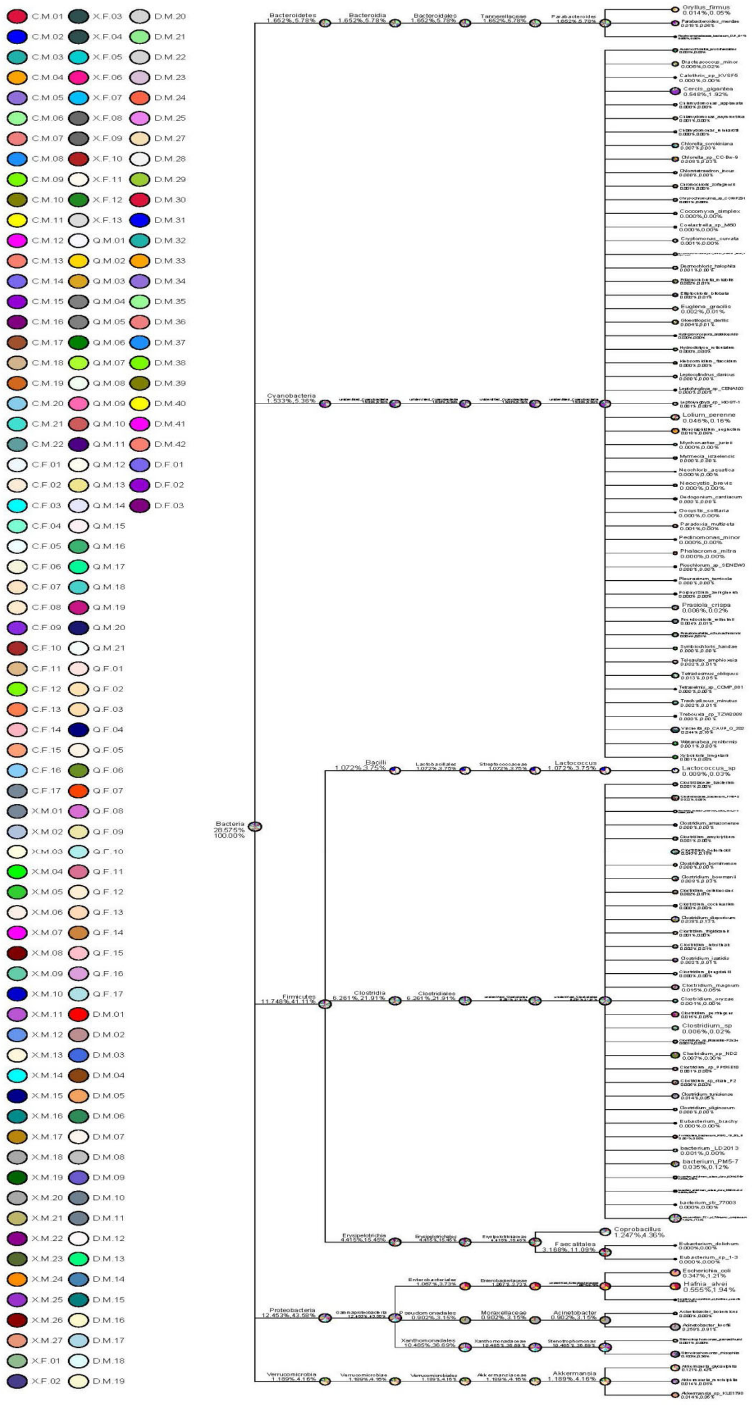

Figure S1. Taxatree in 161 samples. Different colored circles represent different individual, corresponding to the legend on the left; The size of the circle represents the relative abundance of the taxa. The two numbers below the taxa name represent the percentage of relative abundance, the former representing the percentage of all taxa in the sample, and the latter representing the percentage of the taxa in the sample. The classification in red indicates that the classification comment does not exist in the sample, but does exist in other analysis samples.

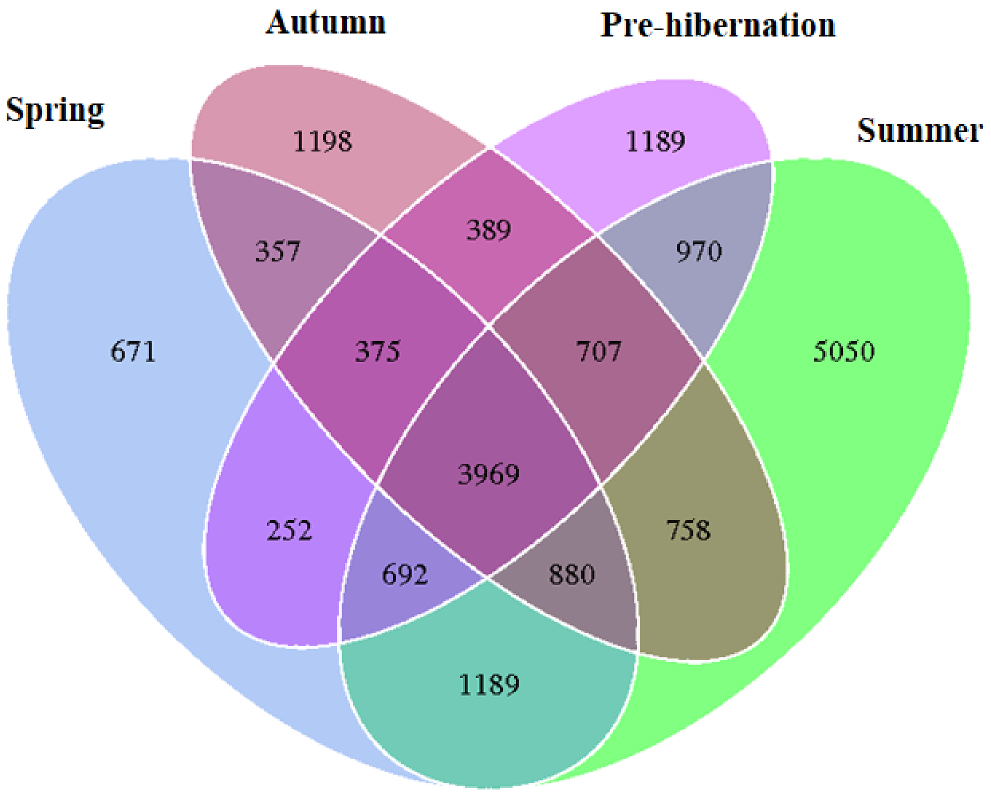

Figure S2. Venn diagram in different seasons. Each circle represents a set of samples. The overlapping part is the number of shared OTUs between different seasons, while the non-overlapping part is the number of its unique OTUs.

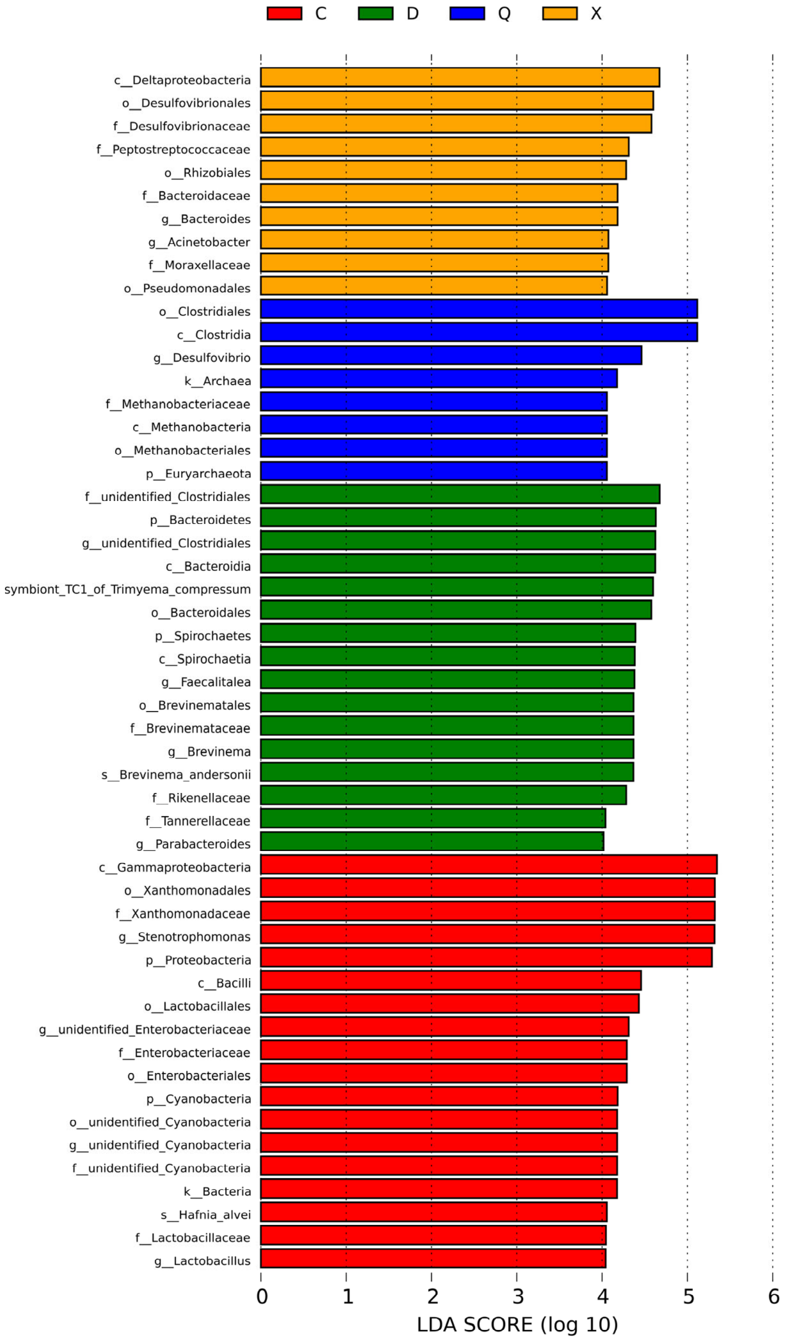

Figure S3. The relative abundance of gut bacterial groups distinguishes frogs of different seasons. The histogram of LDA value distribution showed species with LDA Score greater than the set value (default setting was 4), i.e., biomarkers with statistically significant differences between groups. Shows the species with significant differences in abundance in different groups, and the length of the bar chart represents the impact size of different species (i.e., LDA Score)**.** C: Spring; X: Summer; Q: Autumn; D: Pre-hibernation.

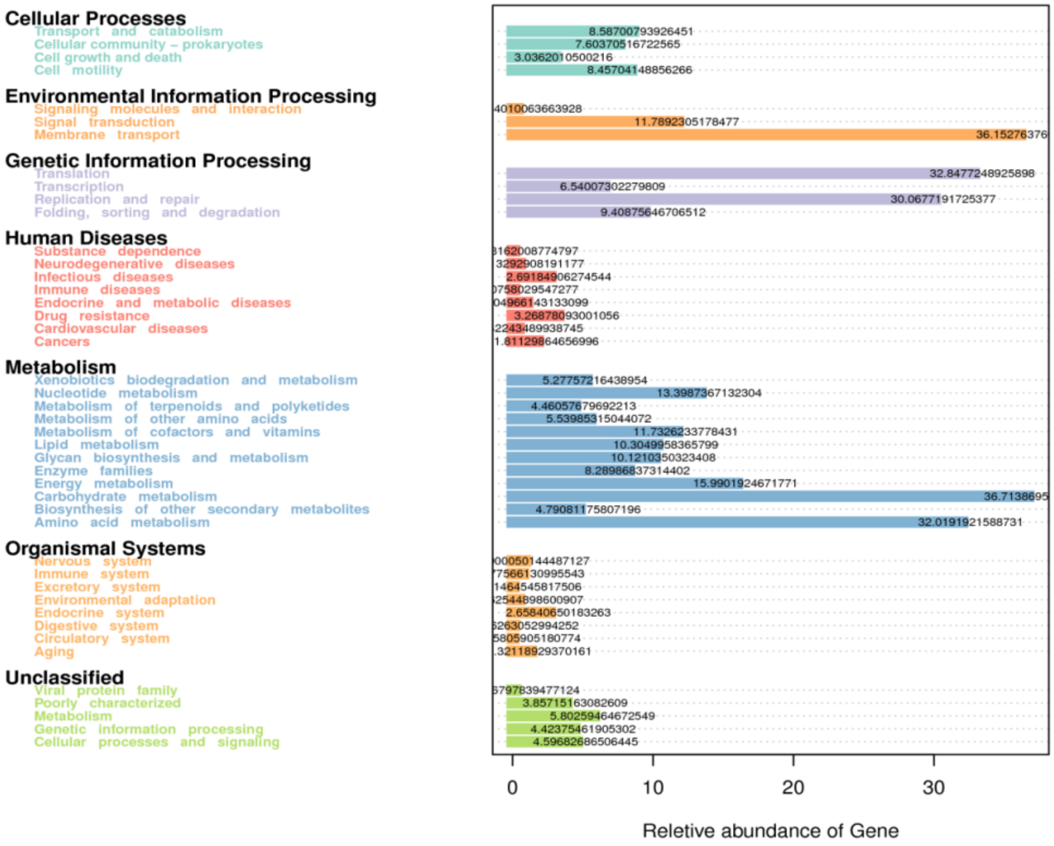

Figure S4. Statistical map of gene prediction results based on KEGG pathway. The predicted function is shown on the left, and the relative abundance of genes expressing this function is shown on the right.

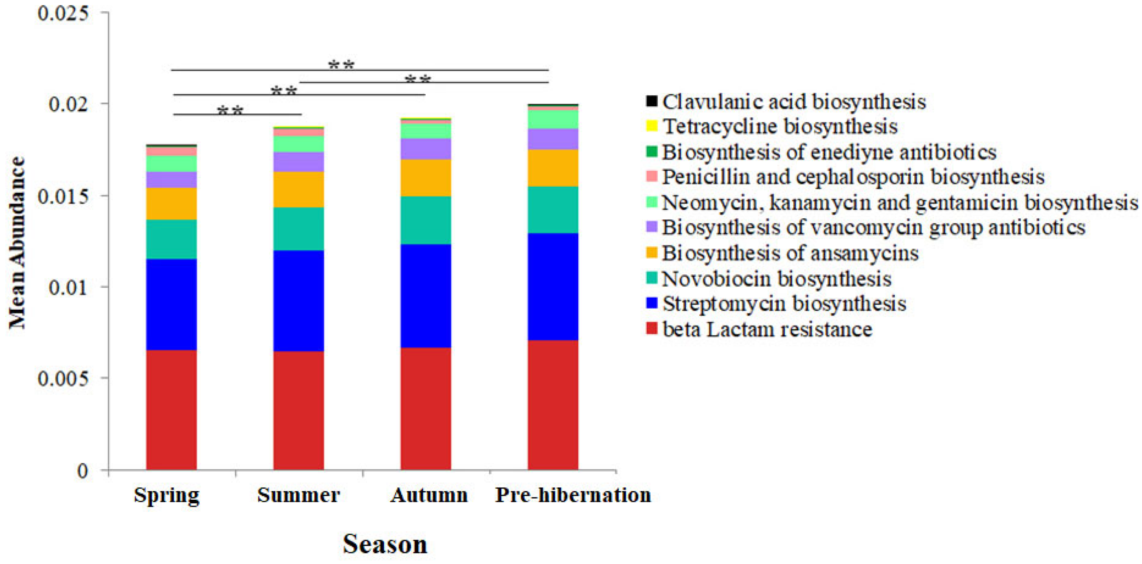

Figure S5. Comparative analysis of the abundance of antibiotic genes between different groups (Wilcoxon rank-sum test; $P < 0.01$**). In the figure, the abscissa is each season, and the ordinate is the relative abundance of antibiotic genes, corresponding to the legend on the right.

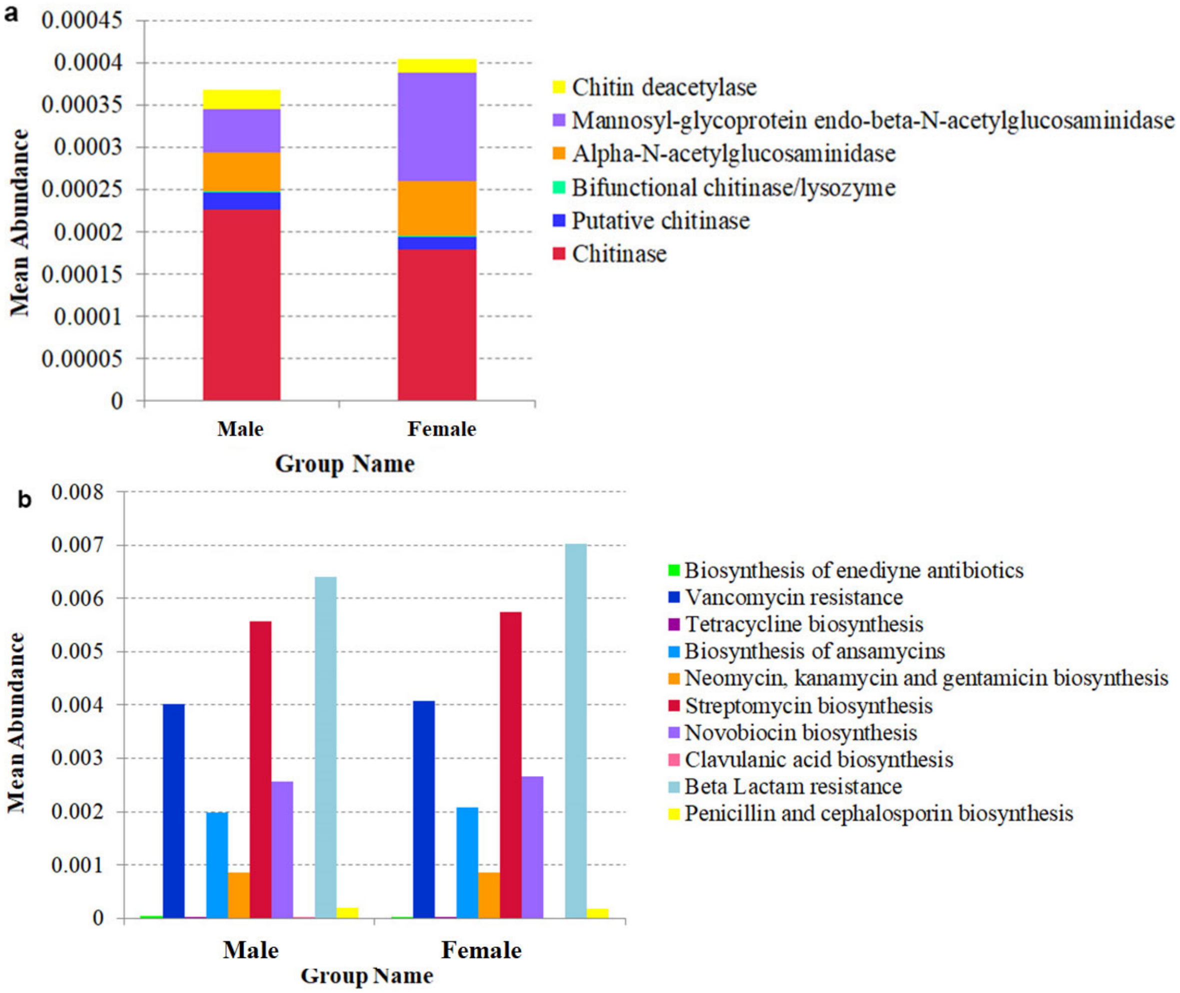

Figure S6. The total abundance of chitinase and antibiotic genes between females and males in autumn. **(a)**: Mean abundance and of Tax4Fun-predicted reads annotated to genes for chitin degrading enzyme. **(b)**: The abundance of antibiotic genes between males and females *F. limnocharis* in autumn.
